# Achieving better maternal and newborn outcomes: coherent strategy and pragmatic, tailored implementation

**DOI:** 10.9745/GHSP-D-13-00030

**Published:** 2013-06-28

**Authors:** Stephen Hodgins

**Affiliations:** a Save the Children, Washington, DC, USA

## Abstract

Maternal and newborn health program effort needs to: shift from mere *contact* to the actual *content* or substance of care; respond better to local context; ensure delivery of all key interventions needed during pregnancy, labor and delivery, and postnatally; and actively monitor performance to manage and improve programs.

With the “Every Woman, Every Child” global initiative^1^ and the “Global Strategy for Women's and Children's Health” implemented under the auspices of the United Nations Secretary-General,^2^ there are now unprecedented political priority and resources available to drive down global maternal and newborn deaths. Although this is certainly welcome, we risk squandering this opportunity if we continue business as usual. Several features of our current efforts are bogging us down, but there is a way forward.
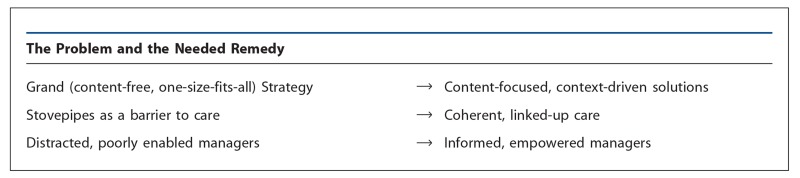


## WHAT IS THE PROBLEM?

### Grand, Relatively Content-Free, One-Size-Fits-All Strategy

During the late 1990s and the early years of the new millennium, contesting camps in ***maternal health*** argued for approaches centered either on provision of: (1) tiered obstetrical services targeting an expected 15% or so of deliveries in which complications might be expected (Emergency Obstetrical Care), or (2) routine care for all deliveries by health care workers with midwifery skills (Skilled Birth Attendance).

Over the past half-decade, the dust from these earlier scuffles has settled; indeed, global leaders in maternal health have been advocating a more nuanced, multipronged approach. However, the one clear message emerging from essentially all global maternal health guidance is some version of “ensure skilled care for every birth”^3^—that is, to increase the proportion of deliveries attended by “skilled birth attendants” (SBAs). Ministries of Health have heard this message loud and clear and are acting upon it, largely to the exclusion of serious attention to the actual care being delivered—even if this was not the intent of global technical leaders promoting Skilled Birth Attendance.

There are important problems both with the particulars of a strategy centered too exclusively on SBA coverage and with the notion that any single service delivery approach will be optimal for all settings. In its effect, the current approach as actually delivered could be characterized as: “Get them in the front door and then trust the clinician.”

Since around 2000, the ***newborn health*** community rapidly established a presence, largely due to the Saving Newborn Lives program, funded by the Bill and Melinda Gates Foundation (BMGF), and to a succession of central projects funded by the U.S. Agency for International Development (USAID) that have included both maternal and newborn health. Without directly challenging the dominant maternal health Grand Strategies, the newborn field has attempted to legitimize a wider domain for action under the rubric of the “household-to-hospital continuum of care,”^4^ with enthusiastic support from at least some in the maternal health community. In practice, however, building on lessons learned from the work of Abhay Bang,^5^ the newborn health community has focused primarily on *community-based* service delivery. This has required some delicacy because the maternal health field has proscribed use of traditional birth attendants (TBAs) at home deliveries. The newborn health field therefore has advocated use of *non-TBA* community health workers (CHWs) to care *only* for the newborn, either by being present for home deliveries or by coming to the home soon after delivery.

#### Contact-Centered

As it plays out at the level of actual service provision, the emphasis in the maternal health field has been on mere *contact*—that is, it has focused on having women deliver in the presence of a skilled birth attendant, simply assuming that the “skilled” provider will do all that is required. This situation has arisen in part due to the almost exclusive reliance on the SBA indicator as the principal proxy measure (or global benchmark indicator) for maternal health program performance, despite abundant evidence that, in many instances, those labeled as “skilled” providers do not have the appropriate skills^6^ and that—whether skilled or not—they often do not do the right things.^7^ So, not surprisingly, we see little, if any, correlation between “skilled birth attendance” and overall maternal or newborn mortality.^8^-^11^

Increasing the proportion of deliveries in the presence of “skilled birth attendants” does not necessarily reduce maternal or newborn mortality.

With its almost exclusive focus on labor and delivery, the current approach as implemented has largely ignored opportunities to achieve better outcomes arising during pregnancy (only paying attention to whether the requisite antenatal *contacts* occur).

Like the prevailing maternal health Grand Strategy, the “postnatal home visits” strategy now being promoted in newborn health^12^ also emphasizes *contact*, assumes that such contact will deliver impact, and gives comparatively little attention to the *content* provided. With an evidence base consisting only of relatively small-scale, intensively supported trials^13^-^14^ and demonstration projects,^5^^,^^15^ the World Health Organization (WHO) and the United Nations Children's Fund (UNICEF) have advised Ministries of Health to develop national programs with CHWs making multiple postnatal home visits.^12^ Many countries have launched such programs; however, to date, there has been no evidence that it is feasible to achieve impact at scale with this approach.

Although global health leaders have increased attention to defining priority content of care in recent years,^16^ there has been little *effective* programmatic attention to content. In maternal health, any attention to content has focused largely on labor and delivery care, notably on management of a particular set of complications (the emergency obstetrical care “signal functions”),^17^ with a nod to “focused antenatal care.”^18^ Under USAID-funded work, there has been some effort given to uterotonic use to prevent postpartum hemorrhage and to “keeping the normal normal.”^19^

Newborn health proponents also have limited their focus largely to the delivery and the early postnatal period, paying particular attention to: kangaroo mother care for low birth weight babies, resuscitation of asphyxiated newborns, community case management of sepsis, and a wider set of clinical and household practices encompassed by the term “essential newborn care.” This has recently expanded somewhat, with more attention now to the use of corticosteroids for threatened preterm labor and a new focus on stillbirths and “intrapartum” (rather than “asphyxia”) deaths. This reframing draws attention to the important opportunities available to influence these outcomes, not only by resuscitating newborns *in extremis* (at the point of death) but also by providing better care *before* they get to that state (either spontaneously or iatrogenically).

Both maternal and newborn health fields still remain quite focused on the “supply side,” with less programmatic attention given to care-seeking and household practices.

Global maternal and newborn health guidance has focused largely on the supply side to the neglect of care-seeking and household practices.

#### Uncontextualized Directives on “How”

So, both the SBA and Postnatal Home Visit strategies have focused mainly on *contact*, either with a health worker of a certain occupational category at the time of delivery or with a community health worker during a postnatal home visit. In both cases, we have prescriptive strategies that *all* are enjoined to adopt, focusing on *how* services are provided (that is, that there be a contact of a certain kind). This is quite different from providing guidance on *what* specific technical content should be delivered (Box 1).

**Box 1: “How” Versus “What”**

**How**

- Tiered complication management services (basic and comprehensive emergency obstetrical care)
- Deliveries by health care workers with midwifery skills
- Community midwives
- Deliveries assisted by traditional birth attendants
- Postnatal home visits by community health workers
- Antenatal risk stratification, with referral of higher risk cases

**What**

- Uterotonics during the third stage of labor
- Corticosteroids for preterm labor
- Antibiotics for sepsis
- Magnesium sulfate for eclampsia
- Chlorhexidine for newborn sepsis prevention
- Resuscitation of asphyxiated newborns
- Intermittent presumptive treatment for malaria
- Early and exclusive breastfeeding
- Tetanus toxoid

It is true that, since epidemiology differs by setting, there does need to be some adaptation or prioritization of the “what” (the technical content) by setting. Nonetheless, the “what” generalizes fairly broadly across settings. This is less so for the “how.” We cannot simply say, “This is an effective strategy. Everyone should be doing it, everywhere.” Randomized trials such as those referenced above^13^^,^^14^ do not help us much here, as we are less interested in the black-and-white question of “Does it work?” than we are in the questions, “*Under what conditions* does it work?” and “Could this be both effective and *implementable at scale in my setting*?”

Conditions vary enormously across (and often within) countries with high maternal and newborn mortality and stillborns. For example, in some countries certain “indirect causes” (such as malaria) are major contributors to poor pregnancy outcomes. Use of specific dangerous practices—whether by TBAs or professional attendants—varies considerably. Access to health facilities and professionals varies greatly due to geography, population density, and availability of human resources (and barriers related to such factors as cost and culture). Settings differ considerably in the robustness of basic support systems (for example, infrastructure and commodity supply chains). So, the strategies likely to be most effective will be those that fit the specific characteristics, drivers, opportunities, and constraints for maternal and newborn health *as they play out locally*.

*How* best to implement effective maternal and newborn health strategies requires attention to local context.

### Stovepipes as a Barrier to Care

#### The Maternal-Newborn Split

To a considerable extent, global technical strategies for maternal and newborn health have moved independently of each other. This defies biology: Until birth, mother and fetus are unequivocally an inseparable dyad, with the well-being of the fetus/newborn fundamentally dependent on whatever happens to the mother. To a large degree, this continues through early newborn life. Furthermore, the opportunities to optimize maternal, fetal, and newborn outcomes arise from much the *same* contacts—through health workers or CHWs during pregnancy, at the time of labor and delivery, and during the postnatal period, either at a health facility or in the home.

Although mother and the fetus/newborn are inextricably linked to each other, global strategies and funding for maternal health and newborn health have separated them.

The conventional clinical division of labor between obstetricians and pediatricians/neonatologists often carries over into maternal-newborn program work (although in many service delivery settings a single provider is responsible for care of both the mother and newborn). Technical leaders in maternal and newborn health sometimes convene and cooperate, but much of the time they spin in their own separate orbits (for example, in their own global plans and strategies, publications, global and national working groups, technical meetings, and conferences).

Donor funding for maternal and newborn health also tends to be siloed in separate funding streams. USAID has bucked this trend to some extent, by combining maternal and newborn health in its central projects (such as the Maternal and Neonatal Health [MNH] program, ACCESS program, and the Maternal, Neonatal and Child Health Integrated Program [MCHIP]). However, despite this management arrangement, *even within these projects* maternal and newborn health still mostly move along independent tracks. In its global projects, BMGF continues to support maternal and newborn work under entirely separate funding mechanisms. (In their *country-level* work, USAID and BMGF do support programs that integrate maternal and newborn health.) And DfID, despite commendable support for maternal health, has to date largely ignored the newborn.

In many cases, in funding and technical agencies and in Ministries of Health, responsibility for maternal and newborn health falls under different management units or technical officers. This can ripple through inappropriately to the service provider level. One consequence of this split has been that technical leaders, in the one camp, have made strategic choices to optimize *their* outcomes of concern without reference to the other. For example, maternal health leaders have, in effect, prohibited programmatic engagement with TBAs or other community-based service providers, because they felt this was of little value for maternal health outcomes, regardless of what benefits there may be for the newborn.^20^

#### Fragmentation Across Programs

Beyond the maternal-newborn gap, a number of important interventions delivered during pregnancy or in the postnatal period are “owned by” other potentially competing programs with their own funding streams, such as:

Pregnancy, labor and delivery, and postnatal interventions falling under other programs (such as HIV, malaria, nutrition) tend not to be well-integrated into maternal and newborn health services.

- Antenatal and postnatal iron supplementation and breastfeeding counseling (nutrition)
- Intermittent presumptive treatment and use of insecticide-treated nets (ITNs) (malaria)
- Tetanus toxoid and hepatitis B immunization (immunization/child health)
- Counseling on postpartum family planning (family planning/reproductive health)
- Syphilis screening and treatment (sexually transmitted infections)
- HIV screening/prevention of mother-to-child transmission (PMTCT) (HIV/AIDS)

Categorical funding and other structural barriers render *antenatal* care (ANC) a kind of programmatic no-man's land, with an absentee landlord (Maternal Health, focusing only on ANC *contacts*) and orphan tenants laboring in other programs. These “orphans” struggle to deliver their interventions through a relatively neglected service delivery channel, or they disengage from ANC altogether and use their own channels, as is often the case with antenatal tetanus toxoid. Similarly, in the limited discussion now heard among global MNH technical leaders on action to be taken in the *postnatal* period, there has been little attention to interventions perceived as belonging to other program areas (such as malaria, immunization, HIV, and nutrition).

Even interventions that can have high impact on reducing mortality in certain settings are rarely championed by technical leaders in MNH, when they are seen as belonging to other programs (for example, ITNs, antenatal iron supplementation, PMTCT, and syphilis screening and treatment). The result of this stove-piping has been that women and newborns are less likely to get these needed (and generally quite simple) interventions and are therefore at higher risk of poor outcomes (see Figure).

**Figure:**
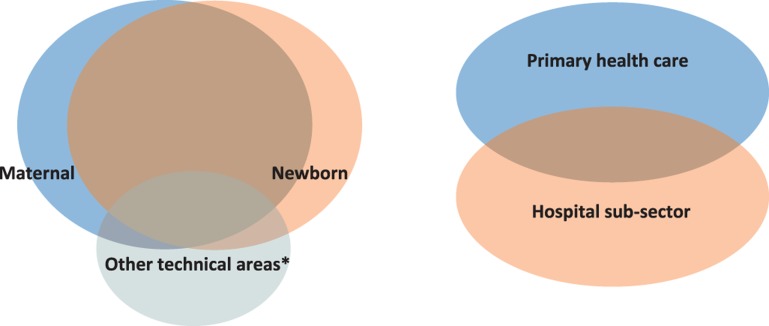
Managing the Overlap: Maternal and Newborn Health Services as an Integral Whole * Examples of other technical areas include nutrition, malaria, and immunization, delivered through antenatal or postnatal care.

#### The Hospital/Primary Health Care Sub-Sector Disconnect

Another unhelpful form of siloing has been between the hospital and primary health care sub-sectors (see Figure). Typically, hospitals are managed under altogether different units of the Ministry of Health than services up to the health center level (which include most antenatal services and, in at least some settings, a significant proportion of institutional deliveries). Sometimes they are managed under altogether different ministries! So, not surprisingly, on-the-ground coordination between these different types of health facility is generally weak, if not non-existent. This contributes to poorly developed referral linkages, with unhappy consequences for many women and newborns experiencing complications that require prompt, effective referral to higher levels of care.

### Distracted, Poorly Enabled Program Managers

As discussed above, regardless of their own judgment on what may be needed, program managers at the country level have been pressed to focus especially on: increasing institutional deliveries and, resources permitting, in-service training for health care workers providing labor and delivery care. On the newborn side, they are given a very short list of interventions and service delivery approaches to “scale up.”

Program managers neither have been encouraged nor have been enabled to *track what is actually happening* when such services are provided—as a basis for taking action to improve effective coverage. Furthermore, responsibility for services or interventions delivered during pregnancy, around the time of delivery, and over subsequent days and weeks is distributed across multiple programs and management units. The result is that no single manager or program is empowered or held accountable to ensure the reliable delivery of the full range of important services at high coverage.

## WHAT IS NEEDED?

### Context-Driven, Content-Focused Solutions

At the global level, we must be careful to avoid being inappropriately prescriptive on service delivery approaches (Grand Strategies). Although those at the global level may not have perceived their guidance in this light, too often this has been its effect at the country level.

Country-level program managers need to be encouraged and supported to determine the most promising strategies for achieving impact *in their settings* by looking at the particular situations they face, including local epidemiology and population distribution, service utilization patterns, barriers to access, availability of resources, and robustness of support systems. Often they can draw useful lessons, or adopt and adapt tools or models, from experience elsewhere, particularly if conditions in those other settings are comparable to their own. But, at the end of the day, program managers will need strategies that fit their own particular circumstances. Strategies, grand or otherwise, are a problem if they are not a good fit with the setting in which they are introduced. We cannot assume fit; we must verify it.

Effective maternal and newborn health strategies are appropriately tailored to the local context.

Rather than being told to all use the same service delivery strategies, program managers need to be encouraged and empowered to determine the most appropriate approaches beginning from where they are.^20^ What opportunities are currently available? For example, if the goal is improved delivery of key postnatal interventions and a relatively high proportion of deliveries in a particular setting currently occur in health facilities, by all means program managers should take advantage of hospital admissions as a platform for providing such interventions (for example, focusing on pre-discharge assessment, counseling about danger signs and essential newborn-care practices, providing hepatitis B immunization, dispensing iron supplements or any other needed supplies). Why ignore this opportunity and, instead, focus efforts on trying to develop a new platform of postnatal home visits by CHWs (particularly when there have been no successful experiences elsewhere implementing such a strategy at scale)?

The whole span of maternal and newborn services comprises a fairly wide range of interventions, delivered over several stages of the life cycle—some on a schedulable basis, some not. Some of this content is simple enough to be delivered by health auxiliaries or CHWs. But other aspects of care require complex skills and support services. Conditions relevant for using a particular strategy vary a great deal by setting. In all these respects, maternal and newborn health is inherently more complex than immunization, for example. We should not be surprised, then, that a greater degree of tailoring to context is needed to effectively deliver maternal and newborn health services to a population.

Our emphasis to date on “skilled birth attendance” has resulted in misdirected program effort. *Where* services are delivered, or *by whom*, do not by themselves drive outcomes. *What is actually done* drives outcomes. The message to country programs needs to change from a focus on *contact* toward *content*—that is, toward the *substance* of care actually delivered. Our focus needs to be on delivery of that substance to all those needing it; “contact” is important only as a means to that end.

Emphasis should be placed on the *actual substance of care* delivered, not on *where* or *by whom* the care is delivered.

In addition to focusing on the specific *positive* practices we want to see, we also need to eliminate common *negative* practices that increase risk of poor outcomes (such as poor asepsis, unsafe labor augmentation, application of fundal pressure, and medically unnecessary procedures with inherent risk, notably elective cesarean delivery).

### Coherent, Linked-Up Care

Pregnant women, newborns, and postpartum mothers require a set of services provided coherently and comprising all needed elements, regardless how the “ownership” of those elements is distributed across programs. It may be unrealistic to tear down all structural, disciplinary, and financial silos, although donors do have scope to combine funding streams and management units. Nevertheless, to the extent that we have to continue to live with such barriers, we need to find ways to mitigate their counterproductive effects on service delivery as experienced by actual beneficiaries.

Where possible, maternal and newborn management units, technical coordination bodies, and funding streams should be merged, and serious efforts made to ensure effectiveness and coherent delivery of the full package of needed interventions, including those currently “belonging” to other programs. As a maternal or newborn health program manager considering, for example, use of bed nets by pregnant women, mothers, and newborns living in malarious areas, it is not good enough to say: “Oh, the malaria people will take care of that with their funds and monitoring systems; that's not our responsibility.” Donors and technical assistance partners need to be part of the solution rather than reinforcing the walls of their silos.

An often-used buzz word in global health today is “integration.” The services that need to be delivered during pregnancy, childbirth, and the postnatal period are crying out for more coherent, integrated effort cutting across current programs and categorical funding streams.

### Informed and Empowered Managers

We can almost never count on our initial plans getting everything right (recalling that “no battle plan survives contact with the enemy”). But once we have begun to implement a plan, if we closely *monitor what is happening practically*—in real time—we can see how our programs or services are actually performing and then, based on this information, take any required action to bring about better performance.

Practical monitoring data directs managers on the actions necessary to improve program performance.

Managers at national and local levels need to be able to track what is actually happening with maternal and newborn services and programs, beyond mere inputs. Although it is generally not possible to track population health status closely, program managers need some reasonable approximation. What is happening with regard to *effective coverage*—in other words, what proportion of all of those in a population requiring a particular service are actually getting it (delivered in a way that its effectiveness is assured)?

Program managers also need to be able to track *key determinants* or drivers of effective coverage (sometimes described as “implementation strength”^22^). For example, if the intervention of interest is adequate case management of newborn sepsis, how is the supply chain for the needed antibiotics performing? What are the stockout rates?

Programs in immunization and tuberculosis have been well-served by a small set of meaningful *program indicators* (for coverage and commodity logistics), tracked regularly through health management information systems, which are actively used as a basis for effective decision-making to address compromised performance. Despite all the measurement work that has been done in maternal and newborn health, effort has largely focused on surveys and special studies, generally conducted as one-offs or—at best—once every 5 years. Such surveys do not meet the need of program managers for ongoing, real-time tracking and management of program performance.

At the health facility level, some efforts *have* been made to implement quality improvement processes or approaches (including use of partographs or checklists, Improvement Collaboratives, Standards-Based Management and Recognition, criterion-based audit, and maternal and perinatal death audits), but these initiatives generally have been too intensive and dependent on external inputs to be implemented effectively at scale on a sustained basis. There have also been efforts by WHO and others to identify a limited number of maternal and newborn content/quality indicators that could be incorporated into routine health management information systems for monitoring at all levels, but this has yet to gather steam (Box 2).

**Box 2: Possible Routine Monitoring Indicators**

- Use of a uterotonic during the third stage of labor, as a percentage of term deliveries
- Intrapartum stillbirths and very early neonatal deaths, as a percentage of term deliveries
- Of all maternal and perinatal deaths, percentage followed up by an audit
- Of health facilities routinely doing deliveries, percentage that have institutionalized death audits for all maternal and perinatal deaths
- Stockout status (for example, any stockout over the previous 3 months) for key program commodities in labor and delivery areas of health facilities (oxytocin, magnesium sulfate, gentamicin, dexamethasone)
- Cesarean deliveries, as a percentage of term deliveries
- Assisted vaginal deliveries, as a percentage of term deliveries
- Of health facilities routinely doing deliveries, percentage in compliance with the baby-friendly hospital initiative

What is needed for maternal-newborn health programs to deliver impact at population scale is a clear shift from just “trusting the clinician” toward *effective monitoring* and *active management* of key aspects of service delivery and program performance at all levels—from the health facility through the Ministry of Health. We need to empower managers, giving them a window on what is actually happening in the services for which they are responsible, as a basis for actively managing them to improve coverage and quality and reduce preventable deaths. And program managers responsible for maternal and newborn health services need to be mandated and held accountable for delivery of all high-impact elements of care at this stage of the lifecycle, including those now falling under the responsibility of other programs.

Our global health community has committed itself to ending preventable child and maternal deaths. Perhaps more than ever, there is a sense of urgency and hope that we really can do something about this continuing tragedy. The key challenge is effective implementation in the real world—no easy task. We need, now, to make sound choices to make that happen.
